# Public attitudes toward larger cigarette pack warnings: Results from a nationally representative U.S. sample

**DOI:** 10.1371/journal.pone.0171496

**Published:** 2017-03-02

**Authors:** Sarah D. Kowitt, Seth M. Noar, Leah M. Ranney, Adam O. Goldstein

**Affiliations:** 1 Department of Health Behavior, Gillings School of Global Public Health, University of North Carolina at Chapel Hill, Chapel Hill, North Carolina, United States of America; 2 School of Media and Journalism, University of North Carolina at Chapel Hill, Chapel Hill, North Carolina, United States of America; 3 Lineberger Comprehensive Cancer Center, University of North Carolina at Chapel Hill, Chapel Hill, North Carolina, United States of America; 4 Department of Family Medicine, University of North Carolina at Chapel Hill, Chapel Hill, North Carolina, United States of America; University of Ottawa, CANADA

## Abstract

A large body of evidence supports the effectiveness of larger health warnings on cigarette packages. However, there is limited research examining attitudes toward such warning labels, which has potential implications for implementation of larger warning labels. The purpose of the current study was to examine attitudes toward larger warning sizes on cigarette packages and examine variables associated with more favorable attitudes. In a nationally representative survey of U.S. adults (*N* = 5,014), participants were randomized to different warning size conditions, assessing attitude toward “a health warning that covered (25, 50, 75) % of a cigarette pack.” SAS logistic regression survey procedures were used to account for the complex survey design and sampling weights. Across experimental groups, nearly three-quarters (72%) of adults had attitudes supportive of larger warning labels on cigarette packs. Among the full sample and smokers only (*N* = 1,511), most adults had favorable attitudes toward labels that covered 25% (78.2% and 75.2%, respectively), 50% (70% and 58.4%, respectively), and 75% (67.9% and 61%, respectively) of a cigarette pack. Young adults, females, racial/ethnic minorities, and non-smokers were more likely to have favorable attitudes toward larger warning sizes. Among smokers only, females and those with higher quit intentions held more favorable attitudes toward larger warning sizes. Widespread support exists for larger warning labels on cigarette packages among U.S. adults, including among smokers. Our findings support the implementation of larger health warnings on cigarette packs in the U.S. as required by the 2009 Tobacco Control Act.

## Introduction

While tobacco use is the leading cause of preventable death and disease in the United States [1], of the 40 million adults that currently smoke cigarettes [2], the vast majority want to quit [3]. One way to increase quit attempts and stimulate smoking cessation may be through health warnings on tobacco products. Because of the frequency with which warning labels are seen by current smokers and their extensive reach to diverse populations, the Institute of Medicine has concluded that prominent health warnings on cigarette packages are “among the most cost-effective forms of public health education available” [4]. In fact, a pack-a-day smoker could potentially be exposed to warning labels over 7,000 times in one year [5], and that exposure may coincide precisely with when a smoker is reaching for a cigarette.

While health warning labels have been required for cigarette packs in the U.S. since 1965, the current health warning labels on packs in the U.S. are largely ineffective, given that they have not been changed for over 30 years (since 1985) [6]. Moreover, the four text-only required warnings are small (around 10% of the package), only appear on the side of packs, and often go unnoticed by current smokers [6]. The 2009 Family Smoking Prevention and Tobacco Control Act required the Food and Drug Administration (FDA) to implement graphic (pictorial) warning labels covering 50% of the front and rear panels of the cigarette package, and similar warnings are required for cigarette advertising at 20% of the advertisement’s area [6, 7]. These larger warning labels have not yet been implemented due to industry litigation that has temporarily halted their implementation [7]. In fact, the final rule was challenged in court and on August 24^th^, 2012, the U.S. Court of Appeals for the District of Columbia Circuit struck down the specific graphic warnings proposed by the FDA, although the size of the required warnings was not challenged [6, 7]. Currently, the FDA is conducting research on new graphic warnings that “aims to support a new rulemaking consistent with the Tobacco Control Act” [7]. Given the delay, eight public health and medical groups filed a lawsuit on October 4, 2016 to force the FDA to issue a final ruling regarding health warnings on cigarette packages [8].

The effectiveness of health warnings on cigarette packs depends on many variables (e.g., content, placement on pack, presence or absence of pictorial, static vs. rotating messages)—one of which is size. Article 11 of the Framework Convention on Tobacco Control (FCTC), a treaty adopted by the World Health Organization to develop universal standards for tobacco control, emphasized the importance of warnings that cover at least 30% of the area of the two principal display panels [9]. The FCTC also noted the effectiveness of larger health warnings, covering more than 50% of the principal display areas, and urged parties to consider larger warnings [9]. These recommendations for larger health warnings have been supported with evidence from a recent review noting the effectiveness of larger health warnings [10] and evaluations of countries that have implemented larger health warnings [5, 11, 12]. Moreover, recent meta-analyses of warning experiments [13] and observational studies [14, 15] have demonstrated a greater impact of pictorial as compared to text-only warnings on a series of cessation-related outcomes; such warnings are virtually always larger and more visible on the cigarette pack.

In Canada, researchers estimated that new warning labels introduced in 2001 (which included implementation of pictorial warnings and increased size from 35 to 50%) decreased adult smoking prevalence between 12 and 20% by 2009 [11]. The authors estimated that if similar warning labels had been implemented in the U.S. in 2012, the number of adult smokers would have been reduced by 5.3 to 8.6 million adults in 2013 [11]. Similarly, a recent analysis estimated that larger pictorial warning labels (covering 50% of the front and back of cigarette packs) in the U.S. could reduce smoking prevalence by 5% over the short term and 10% over the long term. This change would help avert over 650,000 smoking-attributable deaths in the next 50 years, in addition to fewer low-birth weight cases, preterm births, and cases of sudden infant death syndrome [16].

Evidence from other countries also suggests the importance of warning label size in reducing smoking prevalence and increasing quit intentions. When Uruguay increased the size of their graphic warning labels from 50% to 80%, all warning label effectiveness metrics significantly increased (i.e., salience of the warning labels, frequency of thinking about smoking-related harms and quitting, and foregoing cigarettes) [12]. Similarly, in Australia, evaluations of their 2012 implementation of larger, graphic warning labels and plain packs found that the new warnings decreased the appeal of tobacco and cigarette packs among adults [17] and adolescents [18], and increased quit attempts among smokers [19]. It is therefore likely that increasing the size of pack warnings may be an independent factor in warning label effectiveness [10].

Previous research has examined the *effectiveness* of warning label size on smoking-related outcomes, concluding that larger warning sizes are associated with increased knowledge of smoking risks and harms, increased quit intentions and attempts, and potentially decreased smoking prevalence [4–6, 10, 11, 14, 20]. Little research, however, has examined *public attitudes toward* different sizes of warning labels on cigarette packages. Favorable attitudes toward larger pack warning sizes may facilitate policy adoption, which is critical given that larger warnings will only be impactful if they can actually be adopted and implemented. Data on attitudes may also influence what warning size policy-makers will ultimately support, influence policy effectiveness, and identify hard-to-change sub-populations for whom targeted messages or public service announcements may be necessary [21–24]. As one example of the importance of policy support (or lack thereof), part of the failure of the 1998 U.S. Senate tobacco legislation (the “National Tobacco Policy and Youth Smoking Reduction Act”) can be attributed to a lack of broad public support [25]. If research had been conducted examining public attitudes toward the legislation, the findings would have suggested that the public preferred some measures (i.e., youth access restrictions), but not others, and that incremental changes would have been more successful [26]. More recently, when seeking to raise the minimum age to purchase tobacco and “Sensible Tobacco Enforcement”, New York City relied on several strategies to build a broad spectrum of public support (particularly among constituents who were previously unsupportive) [27].

Given the importance of efforts to increase the size of cigarette warnings in the U.S., combined with the lack of research about attitudes towards increased sizes, the purpose of our study was to investigate attitudes of U.S. adults toward larger warning sizes on cigarette packages.

## Methods

### Data source

Data utilized in this research come from a nationally representative telephone survey administered by the Center for Regulatory Research on Tobacco Communication (CRRTC) between September 15, 2014 and May 31, 2015 [28]. The CRRTC phone survey included questions on tobacco regulatory constructs, including tobacco product use and public support for tobacco regulations. Two independent and non-overlapping random digit-dialing frames (landline and cell) were used for sampling, ensuring coverage to approximately 98% of U.S. households. In order to ensure adequate representation among smokers, high-smoking/low-income areas were oversampled. Additionally, cell phone numbers were oversampled to maximize counts of young adults. To be eligible for participation, a telephone number needed to reach a household with an English- or Spanish-speaking resident 18 years of age or older. The sample resulted in 5,014 interviews and a weighted response rate of 42%, which is comparable to other national tobacco surveys [29, 30]. Participation was voluntary and anonymous; informed oral consent was obtained by all participants prior to enrollment in the study. The UNC Chapel Hill Institutional Review Board approved all study procedures including consent procedures. More details on the sampling and data collection procedures can be found elsewhere [28].

### Experimental data

Participants were asked for their attitude toward health warnings of a particular size on cigarette packs. The exact item was: “do you think that the FDA should have a health warning that covers (randomized) % of a cigarette pack?”. One-third of participants were randomized to receive a message that the health warning covers 25% of a cigarette pack, one-third to 50%, and one-third to 75%. Participants were not exposed to actual warnings on cigarette packs but rather were asked their attitude towards warnings of different sizes over the phone. Responses included yes (coded as 1) or no (coded as 0). Participants who refused to answer, reported “do not know”, or reported “no opinion” were characterized as missing and excluded from analysis (n = 238, 4.4% of the sample).

### Measures

#### Demographic characteristics

Demographic characteristics measured included age, sex, race, ethnicity, education, and household poverty status. Age was dichotomized for analyses with categories for young adults (18–24 years old) and adults (25+ years old). We used census categories to define race, which included: White; Black or African American; or other race (including American Indian or Alaska Native; Asian; Pacific Islander; and other). Education was measured with categories for high school or less; and greater than a high school. Household poverty status was classified as above or below the 2014 poverty line based on household size and income reported by participants.

#### Additional tobacco covariates

Additional tobacco covariates used in our study included current smoking status and quit intentions. Current smoking status was measured with two items, asking participants “have you smoked at least 100 cigarettes in your entire life?” and “do you now smoke cigarettes every day, some days, or not at all?” [31]. Participants who reported smoking at least 100 lifetime cigarettes and reported now smoking cigarettes every day or some days were classified as current smokers. Otherwise, participants were classified as not current smokers. Quit intentions were measured with the item “are you planning to quit smoking…” with response options for “within the next month” (coded as 1), “within the next 6 months” (coded as 2), “sometime in the future beyond 6 months” (coded as 3), or “are you not planning to quit” (coded as 4) [32]. This item was only asked of current smokers. For analysis, we reverse coded this item so that the scale of quit intentions ranged from 1–4, where 4 indicates higher quit intentions.

### Data analysis

We used SAS version 9.3 survey procedures to account for the complex survey design and sampling weights [33]. We entered all covariates (i.e., race, ethnicity, age, sex, education, household poverty status, smoking status, quit intentions) and the warning size manipulation simultaneously in a multivariable logistic regression model to estimate odds ratios (ORs) for adults with attitudes supportive of larger health warnings. Additionally, we stratified results by experimental condition to determine variables related to favorable attitudes of different warning sizes, i.e., 25%, 50%, and 75%, and by smoking status to determine variables related to favorable attitudes among current smokers. Only individuals with complete data across all relevant variables were included in the analyses. In our final model for the entire sample, 287 observations (approximately 5.7% of the sample) were deleted because they were missing on some of the explanatory variables. Results include weighted percentages (which adjust for the sampling design, nonresponse rates, and population counts with regards to census region, age, education, gender, ethnicity, phone type, and regional smoking rates) [28], adjusted odds ratios (AOR), and confidence intervals (CI) and may be generalized to all adults in the United States. For all analyses, significance was set at p < 0.05.

## Results

### Participant characteristics

Table 1 provides weighted percentages for our sample (*N* = 5,014). The sample was approximately half female (51.5%); majority over the age of 25 (85.1%); and majority White (68%), non-Latino (85.8%). Participants were educated (57.4% with some college or higher) and mostly above the poverty line (75.3%). Approximately one sixth of participants reported being a current smoker (17.8%) and among current smokers, mean quit intentions were 2.52 (standard error: 0.05) on a scale of 1–4, where 4 indicates higher intentions.

**Table 1:** Unweighted and weighted percentages for demographic and smoking-related variables.

Variable	All adults, unweighted n	All adults, unweighted % or mean	All adults, weighted % or mean
Gender			
Female	2640	52.67	51.48
Male	2372	47.33	48.52
Age			
Adult, 25+ years	4205	83.87	85.14
Young Adult, < 25 years	809	16.13	14.86
Race			
White	3473	69.57	67.99
Black or African American	978	19.59	18.26
Other (American Indian or Alaska Native; Asian; Pacific Islander; and other)	541	10.84	13.75
Ethnicity			
Non-Latino	4568	91.36	85.79
Latino	432	8.64	14.21
Education			
Greater than high school	3241	64.86	57.42
High school or less	1756	35.14	42.58
Household poverty status			
Above the poverty line	3772	75.23	75.29
Below the poverty line	868	17.31	15.93
Missing or refused to answer	374	7.46	8.78
Smoking Status			
Not a current smoker	3856	77.01	82.21
Current smoker	1151	22.99	17.79
Quit intentions, mean	1137	2.46	2.52

Table 2 shows the weighted logistic regression results. Overall, across experimental groups, nearly three-quarters (72%) of adults held favorable attitudes toward all larger cigarette health warnings, including favorable attitudes toward the 25% warning condition (78.2%), the 50% condition (70%) and the 75% condition (67.9%). There were no significant differences in proportion of adults who were in favor of the 75% health warning size vs. the 50% health warning size (AOR: 0.90; 95% CI: 0.66, 1.22; data not shown). Individuals randomized to receive the 50% and 75% health warning label conditions had lower odds of favorable attitudes than individuals randomized to receive the 25% warning label condition (AOR: 0.62; 95% CI: 0.45, 0.84 and AOR: 0.55; 95% CI: 0.42, 0.74, respectively). Males (AOR: 0.65; 95% CI: 0.50, 0.84) and current smokers (AOR: 0.60; 95% CI: 0.45, 0.80) had lower odds of having favorable to larger warnings, regardless of size, than females and non-smokers. Young adults (AOR: 2.25; 95% CI: 1.57, 3.21), African Americans (AOR: 3.13; 95% CI: 2.02, 4.85), individuals of “other” race (AOR: 1.87, 95% CI: 1.27, 2.75), and Latinos (AOR: 2.17, 99% CI: 1.42, 3.34) had higher odds of having favorable attitudes supportive of larger health warning labels, regardless of size, compared to adults over the age of 25 and White, non-Latino respondents.

**Table 2:** Weighted logistic regression results, for the entire sample and current smokers only.

Variable	Adults who were in favor of warnings, entire sample, n (%)	Favorable attitudes, entire sample, Adjusted Odds Ratio (95% CI)	Favorable attitudes, current smokers, Adjusted Odds Ratio (95% CI)
Warning Size			
25%	1216 (78.21)	REF	REF
50%	1161 (70.02)	0.62 (0.45, 0.84)*	0.34 (0.19, 0.60)*
75%	1057 (67.90)	0.55 (0.42, 0.74)*	0.46 (0.26, 0.82)*
Gender			
Female	1964 (76.03)	REF	REF
Male	1468 (67.71)	0.65 (0.50, 0.84)*	0.66 (0.39, 1.13)
Age			
Adult, 25+ years	2804 (69.77)	REF	REF
Young Adult, < 25 years	630 (84.64)	2.25 (1.57, 3.21)*	1.75 (0.92, 3.33)
Race			
White	2172 (65.55)	REF	REF
Black or African American	821 (85.56)	3.13 (2.02, 4.85)*	4.31 (2.28, 8.16)*
Other	424 (83.74)	1.87 (1.27, 2.75)*	1.86 (0.82, 4.24)
Ethnicity			
Not Latino	3073 (69.59)	REF	REF
Latino	350 (86.10)	2.17 (1.42, 3.34)*	3.03 (1.17, 7.84)*
Education			
Greater than high school	2113 (69.41)	REF	REF
High school or less	1306 (75.05)	1.27 (0.95, 1.70)	1.74 (1.05, 2.89)*
Household poverty status			
Above the poverty line	2496 (69.99)	REF	REF
Below the poverty line	672 (79.88)	1.32 (0.84, 2.07)	1.27 (0.75, 2.15)
Missing or refused to answer	266 (74.49)	1.16 (0.73, 1.84)	1.05 (0.35, 3.21)
Smoking Status			
Not a current smoker	2751 (73.62)	REF	N/A
Current smoker	679 (64.87)	0.60 (0.45, 0.80)*	N/A
Quit Intentions	N/A	N/A	1.65 (1.27, 2.14)*

Reference group (REF)

N/A (not applicable)

* p<0.05 level

Among current smokers, the majority of adult smokers reported having a favorable attitude to larger warning label sizes, including favorable attitudes toward the 25% (75.2%), 50% (58.4%) and 75% sizes (61%). Smokers who were African American (AOR: 4.31; 95% CI: 2.28, 8.16), were Latino (AOR: 3.03, 95% CI: 1.17, 7.84), had a high school degree or less (AOR: 1.74, 95% CI: 1.05, 2.89), and had increasing quit intentions (AOR: 1.65, 95% CI: 1.27, 2.14) had higher odds of favorable attitudes to larger health warning labels, regardless of size, compared to White non-Latino smokers, and smokers with greater than a high school degree. After adjusting for covariates, adult smokers randomized to receive the 50% and 75% health warning label conditions had lower odds of having favorable attitudes to larger warnings than smokers randomized to receive the 25% health warning label condition (AOR: 0.34; 95% CI: 0.19, 0.60 and AOR: 0.46; 95% CI: 0.26, 0.82, respectively).

### Stratified by experimental condition

We stratified results by experimental condition to determine variables related to favorable attitudes of each of the warning sizes (Table 3 for the entire sample and Table 4 for smokers only). Among the entire sample, males in the 25% and 50% health warning size conditions had lower odds of having favorable attitudes to larger warning labels than females (AOR: 0.56; 95% CI: 0.36, 0.88 and AOR: AOR: 0.61; 95% CI: 0.38, 0.97, respectively). Young adults in the 25% and 75% health warning size conditions had higher odds of favorable attitudes to larger warning labels than adults over the age of 25 (AOR: 4.59; 95% CI: 2.18, 9.65 and AOR: 1.76; 95% CI: 1.03, 3.00, respectively). Across all three warning label conditions, African Americans had higher odds of favorable attitudes to larger warning labels than White adults (AOR: 5.00; 95% CI: 2.78, 8.97, AOR: 2.88; 95% CI: 1.19, 6.98, and AOR: 2.65; 95% CI: 1.57, 4.48, respectively). Finally, Latinos in the 25% and 50% health warning size conditions had higher odds of having favorable attitudes to larger warning labels than non-Latino adults (AOR: 3.20; 95% CI: 1.50, 6.85 and AOR: 2.44; 95% CI: 1.05, 5.68, respectively).

**Table 3:** Weighted logistic regression results, stratified by experimental condition.

Variable	Favorable attitudes among individuals randomized to 25% condition, Adjusted Odds Ratio (95% CI)	Favorable attitudes among individuals randomized to 50% condition, Adjusted Odds Ratio (95% CI)	Favorable attitudes among individuals randomized to 75% condition, Adjusted Odds Ratio (95% CI)
Gender			
Female	REF	REF	REF
Male	0.56 (0.36, 0.88)*	0.61 (0.38, 0.97)*	0.77 (0.53, 1.13)
Age			
Adult, 25+ years	REF	REF	REF
Young Adult, < 25 years	4.59 (2.18, 9.65)*	1.75 (0.96, 3.19)	1.76 (1.03, 3.00)*
Race			
White	REF	REF	REF
Black or African American	5.00 (2.78, 8.97)*	2.88 (1.19, 6.98)*	2.65 (1.57, 4.48)*
Other	1.65 (0.88, 3.09)	1.93 (0.97, 3.85)	2.31 (1.18, 4.51)*
Ethnicity			
Not Latino	REF	REF	REF
Latino	3.20 (1.50, 6.85)*	2.44 (1.05, 5.68)*	1.66 (0.85, 3.27)
Education			
Greater than high school	REF	REF	REF
High school or less	1.14 (0.63, 2.07)	1.15 (0.71, 1.87)	1.33 (0.85, 2.08)
Household poverty status			
Above the poverty line	REF	REF	REF
Below the poverty line	1.14 (0.63, 2.07)	0.81 (0.36, 1.82)	3.32 (1.79, 6.15)*
Missing or refused to answer	0.80 (0.35, 1.79)	1.73 (0.77, 3.92)	1.02 (0.54, 1.94)
Smoking Status			
Not a current smoker	REF	REF	REF
Current smoker	0.70 (0.43, 1.14)	0.48 (0.29, 0.77)*	0.63 (0.39, 1.02)

Reference group (REF)

* p<0.05 level

**Table 4:** Weighted logistic regression results among current smokers, stratified by experimental condition.

Variable	Favorable attitudes among individuals randomized to 25% condition, Adjusted Odds Ratio (95% CI)	Favorable attitudes among individuals randomized to 50% condition, Adjusted Odds Ratio (95% CI)	Favorable attitudes among individuals randomized to 75% condition, Adjusted Odds Ratio (95% CI)
Gender			
Female	REF	REF	REF
Male	0.58 (0.24, 1.39)	1.04 (0.46, 2.35)	0.65 (0.24, 1.78)
Age			
Adult, 25+ years	REF	REF	REF
Young Adult, < 25 years	1.94 (0.52, 7.31)	2.69 (1.07, 6.73)*	0.43 (0.15, 1.20)
Race			
White	REF	REF	REF
Black or African American	2.34 (0.90, 6.08)	14.05 (5.97, 33.06)*	2.27 (0.87, 5.94)
Other	1.59 (0.29, 8.75)	2.20 (0.62, 7.76)	2.00 (0.56, 6.89)
Ethnicity			
Not Latino	REF	REF	REF
Latino	1.81 (0.25, 13.27)	6.20 (1.76, 21.83)*	2.39 (0.53, 10.82)
Education			
Greater than high school	REF	REF	REF
High school or less	3.48 (1.47, 8.23)*	0.94 (0.45, 1.96)	2.00 (0.76, 5.23)
Household poverty status			
Above the poverty line	REF	REF	REF
Below the poverty line	0.34 (0.15, 0.78)*	1.34 (0.54, 3.32)	6.74 (2.48, 18.28)*
Missing or refused to answer	0.42 (0.07, 2.67)	2.49 (0.36, 17.48)	1.07 (0.27, 4.27)
Quit Intentions	1.69 (1.12, 2.54)*	1.60 (1.13, 2.26)*	1.65 (0.93, 2.92)

Reference group (REF)

* p<0.05 level

Among smokers only (Table 4), individuals with increasing quit intentions in the 25% and 50% cigarette health warning size conditions had higher odds of having favorable attitudes to larger warning labels (AOR: 1.69; 95% CI: 1.12, 2.54 and AOR: 1.60; 95% CI: 1.13, 2.26, respectively). Smokers whose household income fell below the poverty line in the 25% had lower odds of having favorable attitudes to larger warning labels than smokers whose income fell above the poverty line (AOR: 0.34, 95% CI: 0.15, 0.78); whereas smokers whose household income fell below the poverty line in the 75% condition had higher odds of having favorable attitudes supportive of larger cigarette warning labels (AOR: 6.74, 95% CI: 2.48, 18.28). Finally, young adults, African Americans, and Latinos in the 50% cigarette warning size conditions had higher odds of having favorable attitudes to larger warning labels than adults over the age of 25 and White, non-Latino adults (AOR: 2.69, 95% CI: 1.07, 6.73, AOR: 14.05; 95% CI: 5.97, 33.06 and AOR: 6.20; 95% CI: 1.76, 21.83, respectively).

## Discussion

Results from our study indicate that the majority of U.S. adults—including smokers—have favorable attitudes toward larger health warning labels on cigarette packs. Indeed, it is notable that nearly three-quarters (72%) of adults had favorable attitudes toward warning labels as large as 75% of a cigarette pack, and even larger percentages of adults had favorable attitudes toward warning labels covering at least 50% of a cigarette pack. Additionally, we found that young adults, racial / ethnic minorities, and non-smokers were more likely to have attitudes in favor of larger warning sizes, whereas among smokers only, males and those with lower quit intentions were less likely to have attitudes in favor of larger sizes. These results are consistent with a recent study that examined attitudes about graphic warnings among US adults [34].

As of 2014, more than 134 countries or jurisdictions have required cigarette health warning labels to cover at least 30% of the package, and over 60 countries or jurisdictions have required cigarette health warning labels to cover at least 50% of the cigarette package. Moreover, 6 countries (Thailand, Australia, Uruguay, Brunei, Canada, and Nepal) have required cigarette health warning labels to cover 75% or more of the cigarette package [35]. The Tobacco Control Act of 2009 required pictorial warning labels to cover 50% of the front and rear panels of cigarette packages in the U.S. [7]. In the 2010 and 2011 proposed and final rulings regarding this 50% requirement, the FDA cited extensive evidence in support of this size requirement, including FCTC recommendations, evaluations of countries that have implemented larger health warnings (e.g., Canada, New Zealand, Australia), and experiments manipulating the size of warning labels [36, 37]. However, as of 2014, the U.S. was one of 50 countries that had not implemented pictorial warnings and larger warnings on the front or back of cigarette packages [35], and that remains the case today in 2017.

Our study suggests that if the provision for larger warning labels on cigarette packages is implemented as required by federal law, there would be widespread support among the American public, including among smokers who would be most affected by the new warnings [38]. The extent of favorable attitudes toward warnings that we observed are consistent with, if not higher than, attitudes for other tobacco control policies. For instance, Rose et al. examined national attitudes for enacted and potential point-of-sale tobacco control policies and found moderate to poor levels of support (less than 50%) for a variety of point-of-sale policies, such as advertising restrictions, product bans, and promotion restrictions [39]. Thus, compared to other tobacco control policies that have been proposed or implemented, our study demonstrates strong support. The findings about smokers (i.e., 61% of smokers showing favorable attitudes toward a warning label of 75% size) is particularly important given that it is substantially higher than levels of favorable attitudes found among smokers for a variety of other tobacco control policies [39], and smokers are usually more opposed to tobacco control policies than non-smokers [39–41].

Research from public policy and agenda setting theory suggests that public attitudes are an important factor in policy adoption, implementation, and effectiveness [23, 24]. Indeed, there are several examples from tobacco regulatory science in which public attitudes were influential in enacting tobacco control policies at the state [42] and federal level [25]. For instance, policy-makers may be more likely to vote for tobacco control legislation if they perceive constituent support for the proposed legislation [22]. Research also suggests that public attitudes may play a role in policy effectiveness. For instance, in a study of attitudes toward anti-tobacco policy among California youth, favorable attitudes were associated with advocacy behaviors, such as asking someone not to smoke [21]. Furthermore, reactance theory suggests that if individuals perceive certain policies to be too restrictive or limiting of personal autonomy, they may respond by (at best) ignoring such policies or (at worst) adopting an opposing attitude, and research finds that reactance may indeed reduce the effectiveness of pictorial warnings [43]. While our study provides evidence that the majority of Americans have favorable attitudes toward larger cigarette health warnings, future research (both qualitative and quantitative) could be particularly useful in examining reasons why individuals may or may not favor larger warning sizes and the effects of implementing larger warning sizes among smokers who are and are not in favor of such changes.

No significant differences existed between levels of support for warning labels that cover 50% of the cigarette pack and those that cover 75% of the cigarette pack. This finding is important given that a growing body of research has established that warning label effectiveness may increase with size and that warning labels that cover 75% of a cigarette pack or greater may be more impactful than those that only cover 50%. For instance, in Canada, researchers found that increasing the size of pictorial warnings on cigarette packages from 50% to 75%, 90%, and 100% enhanced their effectiveness among adult smokers, youth smokers, and vulnerable youth non-smokers [44, 45]. Although the 2009 Tobacco Control Act required warning labels to cover at least 50% of a cigarette package, the FDA has regulatory authority to increase the size of warning labels to higher levels. Since public health benefits may increase with warning label size and U.S. adults may equally support larger warning labels, future tobacco control policy in the U.S. may consider implementation of warning labels of 75% size. As research suggests that each major strengthening of warning labels increases their effectiveness, after implementing warnings that are 50% for a period of time, further increasing the size to 75% should have additional impact [14, 15].

Lastly, we found favorable attitudes for larger warning sizes to mirror some demographic findings for other tobacco control policies. Specifically, we found women, young adults, racial / ethnic minorities (i.e., African Americans and Latinos), and non-smokers to be more in favor of larger warning labels, as has been documented for other policies [39–41]. Given that the prevalence of current smoking is higher among young adults and racial / ethnic minorities, the fact that our study observed more favorable attitudes among these demographics is notable [46]. It is possible that since young adults and racial / ethnic minorities have lower quit rates (i.e., they are less successful in quitting smoking) [46], they would be more likely to favor tobacco control efforts, such as larger warning sizes, that would benefit future quit attempts.

While smokers were overall less likely to view larger health warnings favorably, we found that several sub-groups of smokers (racial / ethnic minorities, individuals with higher quit intentions, individuals with a high school degree or less) were more supportive of larger health warnings, a finding consistent with previous research on public opinion of tobacco control policies [39] and one study of graphic warnings in the U.S. [34]. Contrary to some research that suggests that older adults may be more supportive of tobacco control policies and conflicting evidence regarding the role of education and SES [39], we found young adults to be more in favor of larger warning sizes and little consistent relationship between education and support for larger warning labels.

### Limitations

Our study had several limitations. First, participants heard about potentially larger cigarette pack warnings, rather than actually seeing the warnings, which could have influenced attitudes toward the cigarette warnings. Second, our experiment did not inform participants whether the pack warnings would be text-only or pictorial/graphic and where they would appear on the pack. Third, we did not inform participants about the size or placement requirements for current cigarette pack warnings. Fourth, our study only assessed one aspect (i.e., size) of the Tobacco Control Act warning label requirements. Future research may also examine size in addition to the requirement for pictorial warning labels. Last, some of the estimates in the models stratified by warning size condition and/or smoking status were unstable due to small cell counts. Despite these limitations, our study provides nationally representative data largely illustrating positive public attitudes toward larger health warning labels on cigarette packages.

## Conclusions

Our study demonstrated that the majority of U.S. adults view large warning labels on cigarette packages favorably, with over two-thirds favoring warning labels that cover 75% of a cigarette pack and increasing support for warning labels that cover 50%. Additionally, we found that young adults, African Americans, individuals classified as low poverty status, and smokers intending to quit were more likely to hold favorable attitudes toward larger warning sizes, whereas males and current smokers (including those not intending to quit) were less likely to hold favorable attitudes toward larger warning sizes. Tobacco control policies in support of larger warning labels are likely to be strongly supported by the American public. Future research may be particularly useful in explaining why individuals may be in favor of these larger warning sizes and the influence of such attitudes on responses to warnings and cessation-related behavioral changes.
